# Development and validation of a nomogram predicting the overall survival of stage IV breast cancer patients

**DOI:** 10.1002/cam4.1224

**Published:** 2017-10-04

**Authors:** Shunrong Li, Jianli Zhao, Liling Zhu, Fengxi Su, Kai Chen

**Affiliations:** ^1^ Guangdong Provincial Key Laboratory of Malignant Tumor Epigenetics and Gene Regulation Sun Yat‐Sen Memorial Hospital Sun Yat‐Sen University Guangzhou China; ^2^ Breast Tumor Center Sun Yat‐sen Memorial Hospital Sun Yat‐sen University Guangzhou Guangdong 510120 China; ^3^ Department of Biostatistics Yale School of Public Health Yale University New Haven Connecticut 06511

**Keywords:** Breast cancer, NCDB, nomogram, overall survival, prediction, prognosis, stage IV

## Abstract

This study aimed to develop a nomogram to predict the overall survival (OS) of stage IV breast cancer patients. We searched the National Cancer Database (NCDB) for stage IV breast cancer patients diagnosed between 2010 and 2013. Predictors of OS were identified and a nomogram was developed and validated using concordance index (C‐index), calibration plots, and risk group stratifications. A total of 7199 patients from the NCDB were included in the study. With a median follow‐up of 25.7 months, the 1‐year and 3‐year OS rates were 80.6% and 52.5%, respectively. Race, age, comorbidity status, T‐stage, grade, ER/PR/Her2 status, the presence of lung/liver/brain metastasis, surgery, radiotherapy, and chemotherapy were significantly associated with OS. The developed nomogram had a C‐index of 0.722 (95% CI 0.710–0.734) and 0.725 (95% CI 0.713–0.736) in the training and the validation cohorts, respectively. The predicted survival using the nomogram is well correlated with actual OS. The nomogram was able to stratify patients into different risk groups, among which the survival benefit of local therapy varied. We developed a nomogram to predict the overall survival of stage IV breast cancer patients. Prospectively designed studies with international collaborations are needed to further validate our nomogram.

## Introduction

Breast cancer is a heterogeneous disease with different subtypes, each having different prognoses. Accurately estimating the prognosis of each patient may not only benefit clinical decision‐making but also inform the individualized design of surgical follow‐up surveillance plans. For early‐stage breast cancer, several risk prediction models (RPM) have been developed and widely validated 1, including NPI 2, Adjuvant! 3, Oncotype Dx 4, and Mammaprint 5, etc. However, no RPM has been widely accepted for Stage IV breast cancer patients. The TBCRC 013 study 6 suggested that the 21‐Gene recurrence score has prognostic value in stage IV breast cancer patients. However, there were more clinicopathological features reported to be significantly associated with survival in these patients, such as progesterone receptor (PR) positivity 7, molecular subtype 8, tumor grade 8, and metastatic patterns 9. There are no prognostic models with all clinicopathological features for survival prediction of stage IV breast cancer patients. The National Cancer Database (NCDB) is hospital based, and the participating centers are required to submit data to the database, and the data covers approximately 70% of cancer patients in United States. In this study, we used the NCDB to investigate the prognostic factors of survival in stage IV breast cancer patients and developed a nomogram using these prognostic factors for survival predictions. The aim of this study was to develop a prognostic model that could be used for individualized risk assessment of stage IV breast cancer patients.

## Method

We searched the NCDB database for eligible patients. Data including the site of metastasis (bone, brain, liver and lung) and ER/PR/Her2 status are only available after 2010. In this study, we only included patients with critical data available. The detailed inclusion and exclusion criteria are listed as follows:

Inclusion
  1. Female breast cancer.
  2. Diagnosed between 2010 and 2013.
  3. American Joint Committee on Cancer (AJCC) stage IV.
  4. Confirmed pathology.
  5. No prior diagnosis of breast cancer.

Exclusion criteria
  1. Follow‐up months equal to 0.
  2. Phyllodes tumor.
  3. Unknown bone, liver, lung or brain metastatic status.
  4. Unknown ER, PR, or HER2 status
  5. Unknown race, tumor grade, surgery, radiotherapy, and chemotherapy.

This is an epidemiological study using de‐identified data from the NCDB registries. Therefore, consent for patient participation and study publication is not required. The study approval was waived by the ethical committee of Sun Yat‐sen Memorial Hospital based on our institutional policy. This study was reported using the STARD statement guidelines 10.

The following data were collected for each patient: year of diagnosis, age, race, county, Charlson‐Deyo score, laterality, primary tumor site, tumor grade, T‐stage, histology, estrogen receptor (ER) status, progesterone receptor (PR) status, HER2 status, metastatic sites (bone/brain/lung/liver), primary surgery categorization, radiation therapy status, chemotherapy, survival month, and overall survival status. Charlson‐Deyo score is a weighted score derived from the sum of score for each of the comorbid conditions. Higher score indicates more comorbid conditions. (http://ncdbpuf.facs.org/) Patients were categorized into two age groups based on their age at diagnosis (≤60 years, >60 years), as the median age of the study population was close to 60. Radiation therapy (RT) was divided into two categories (with RT and without RT).

### Statistical analysis

We assigned the eligible patients into the training and validation study cohort, respectively (Detailed in Data S1). We conducted a descriptive analysis of the baseline clinicopathological features of the included patients and used the Chi‐square test to compare the characteristics of patients between the training and validation cohort. The median follow‐up was calculated as the median observed survival time of the entire population. Overall survival (OS) was measured as the time from diagnosis to death due to any causes. The cumulative OS rates were estimated using Kaplan–Meier analysis.

We used the unadjusted Cox regression model as a univariate analysis to screen for prognostic factors of overall survival. Factors determined to be significant by the competing‐risk analysis were incorporated into the Cox proportional hazard regression as a multivariate analysis.

We used the Cox regression model and the “rms” package in R to develop an OS prediction nomogram with 1‐year and 3‐year OS as the endpoints. To evaluate the discriminative ability of the nomogram, we used the Harrell's concordance index (C‐index) 11 with a 95% CI. To assess the accuracy of the nomogram, we used calibration plots to visualize the agreement between the predicted and actual 1‐year and 3‐year OS. All *P‐*values were two‐sided. *P‐*values less than 0.05 were considered statistically significant. The statistical analysis was performed using Stata/MP, version 13.0 (StataCorp LP, College Station, TX, USA) and R.

## Results

### Baseline clinicopathological features

**Figure 1:**
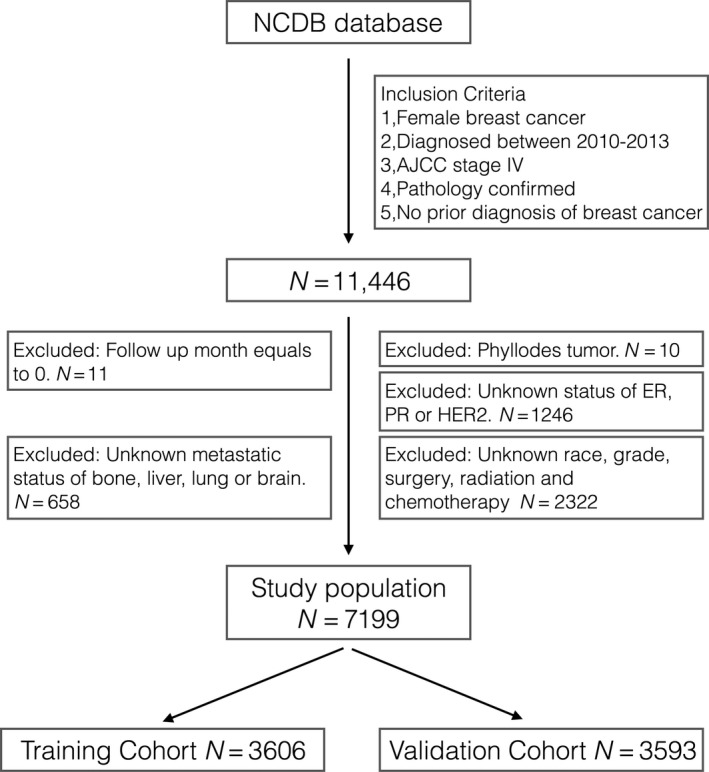
We applied the inclusion and exclusion criteria to NCDB database and enrolled 7199 patients as the study population.

A total of 7199 patients were included from the NCDB database. The number of patients being excluded at each step during the patient selection was summarized in Figure 1.

The clinicopathological features of the patients are summarized in Table 1. The median age (25th–75th percentile) was 58 (49–67) years old. There were 62.7%, 24.8%, 6.0%, and 26.1% of the patients having bone, liver, brain, and lung metastasis, respectively. The median follow‐up was 25.7 months. The 1‐year and 3‐year OS rates were 80.6% and 52.5%, respectively. There were no significant differences between the training and validation cohort.

**Table 1:** Clinicopathological features of the included patients

	Study population				
	Training		Validation		*P* a
	N	%	N	%	
Year of diagnosis					
2010	851	23.60	870	24.21	NS
2011	962	26.68	991	27.58	
2012	854	23.68	884	24.60	
2013	939	26.04	848	23.60	
County type					
Metropolitan	2991	82.95	2950	82.10	NS
Nonmetropolitan/unknown	615	17.05	643	17.90	
Race					
White	2839	78.73	2858	79.54	NS
African American	614	17.03	593	16.50	
Others	153	4.24	142	3.95	
Age group					
<=60	2046	56.74	1987	55.30	NS
>60	1560	43.26	1606	44.70	
Laterality					
Left	1798	49.86	1803	50.18	NS
Right	1778	49.31	1749	48.68	
Others^b^	30	0.83	41	1.14	
Primary site					
Nipple/central portion	219	6.07	243	6.76	NS
UIQ	274	7.60	243	6.76	
LIQ	147	4.08	146	4.06	
UOQ	979	27.15	1001	27.86	
LOQ	243	6.74	217	6.04	
Overlapping/unknown	1744	48.36	1743	48.51	
Charlson/Deyo score					
0	2950	81.81	2923	81.35	NS
1	530	14.70	516	14.36	
2	126	3.49	154	4.29	
Histology					
IDC	2751	76.29	2715	75.56	NS
ILC	346	9.60	336	9.35	
NOS/others	509	14.12	542	15.08	
T‐stage					
T0–T1	491	13.62	479	13.33	NS
T2	856	23.74	875	24.35	
T3	418	11.59	387	10.77	
T4	522	14.48	528	14.70	
Tx	1319	36.58	1324	36.85	
N‐stage					
N0	372	10.32	399	11.10	NS
N1	712	19.74	714	19.87	
N2	522	14.48	532	14.81	
N3	514	14.25	459	12.77	
Nx	1486	41.21	1489	41.44	
Grade					
I	257	7.13	244	6.79	NS
II	1441	39.96	1459	40.61	
III	1908	52.91	1890	52.60	
ER					
Negative	972	26.96	942	26.22	NS
Positive	2634	73.04	2651	73.78	
PR					
Negative	1449	40.18	1384	38.52	NS
Positive	2157	59.82	2209	61.48	
Her‐2					
Negative	2668	73.99	2663	74.12	NS
Positive	938	26.01	930	25.88	
Bone metastasis					
No	1351	37.47	1332	37.07	NS
Yes	2255	62.53	2261	62.93	
Lung metastasis					
No	2672	74.10	2651	73.78	NS
Yes	934	25.90	942	26.22	
Liver metastasis					
No	2701	74.90	2736	76.15	NS
Yes	905	25.10	857	23.85	
Brain metastasis					
No	3382	93.79	3388	94.29	NS
Yes	224	6.21	205	5.71	
Breast surgery					
No_surgery	1401	38.85	1427	39.72	NS
Bcs	616	17.08	620	17.26	
Mastectomy	1589	44.07	1546	43.03	
Radiation therapy					
No	2123	58.87	2132	59.34	NS
Yes	1483	41.13	1461	40.66	
Chemotherapy					
None	1360	37.71	1358	37.80	NS
Single‐agent chemotherapy	492	13.64	504	14.03	
Multiagent chemotherapy	1754	48.64	1731	48.18	

ER, estrogen receptor; PR, progesterone receptor; HER2, human epidermal growth factor receptor 2; LIQ, lower‐inner quadrant; LOQ, lower‐outer quadrant; UIQ, upper‐inner quadrant; UOQ, upper‐outer quadrant; BCS, breast‐conserving surgery; IDC, infiltrating ductal carcinoma; ILC, infiltrating lobular carcinoma; NOS, non otherwise specific.

a Chi‐square test.

b Bilateral/side unspecified/unknown included.

### Screen for prognostic factors for OS

Prognostic factors including year of diagnosis, tumor location (Quadrants), and tumor laterality are theoretically not associated with survival, and were excluded from this analysis. We used unadjusted Cox regression and observed that race (White vs. African American), age (≤60 years vs. >60 years), Charlson score (1 or 2 vs. 0), T‐stage, tumor grade(III vs. I), ER/PR/HER2 status, lung metastasis (Yes vs. No), liver metastasis (Yes vs. No), brain metastasis (Yes vs. No), breast surgery(BCS/Mastectomy vs. No), radiation therapy (Yes vs. No), and chemotherapy (Multiagent vs. None) were significantly associated with OS. The presence of bone metastasis, N‐stage (N2 vs. N0), and histology (IDC, ILC, Others) were not associated with OS (Table S1). We noticed that some subgroup of patients had similar OS, and therefore we combined them as one category in the multivariate analysis, including T0‐1 and T2, T4 and Tx, Grade I and II, no chemotherapy and single‐agent chemotherapy, BCS and mastectomy. In the multivariate analysis, all of these factors were significantly correlated with OS (Table 2).

**Table 2:** Multivariate Cox regression

Features	HR (95% CI)	*P*
Race		
White	1	
African American	1.14 (1.01–1.29)	0.032
Others/unknown	0.59 (0.44–0.78)	<0.001
Age group		
<=60	1	
>60	1.22 (1.10–1.34)	<0.001
Charlson/Deyo score		
0	1	
1	1.37 (1.21–1.55)	<0.001
2	2.04 (1.63–2.56)	<0.001
T‐stage		
T0–T2	1	
T3	1.28 (1.08–1.51)	0.004
T4/Tx	1.51 (1.32–1.72)	<0.001
Grade		
I–II	1	
III	1.26 (1.13–1.40)	<0.001
ER		
Negative	1	
Positive	0.64 (0.56–0.74)	<0.001
PR		
Negative	1	
Positive	0.61 (0.54–0.70)	<0.001
HER‐2		
Negative	1	
Positive	0.52 (0.46–0.59)	<0.001
Lung metastasis		
No	1	
Yes	1.34 (1.20–1.49)	<0.001
Liver metastasis		
No	1	
Yes	1.54 (1.38–1.72)	<0.001
Brain metastasis		
No	1	
Yes	1.62 (1.36–1.94)	<0.001
Breast surgery		
No_surgery	1	
Surgery	0.74 (0.65–0.84)	<0.001
Radiation therapy		
No	1	
Yes	0.87 (0.78–0.96)	0.007
Chemotherapy		
None/single‐agent chemotherapy	1	
Multiagent chemotherapy	0.72 (0.65–0.80)	<0.001

ER, estrogen receptor; PR, progesterone receptor; HER‐2, human epidermal growth factor 2.

### Nomogram development and validation

**Figure 2:**
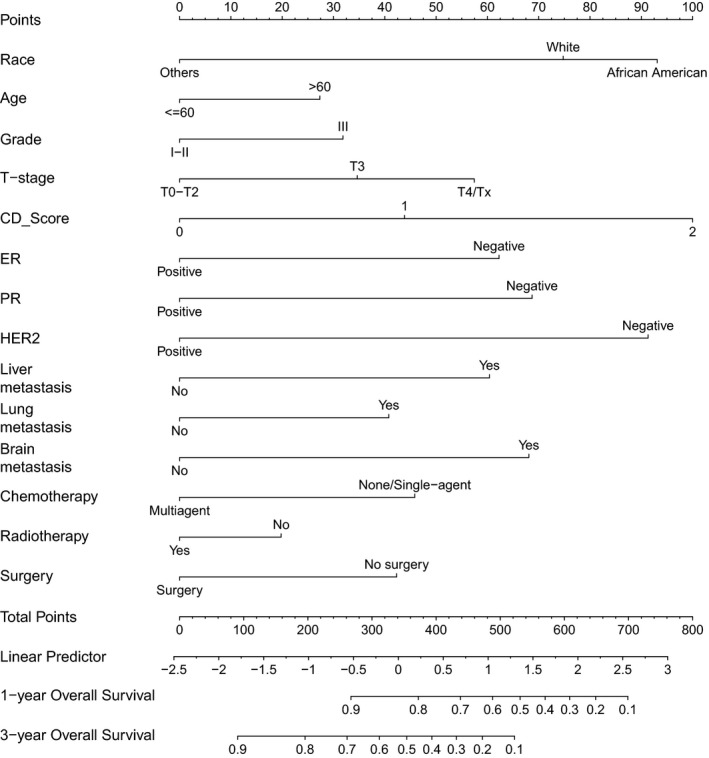
Nomogram to predict the 1‐year and 3‐year overall survival. For each patient, we calculated the points of the corresponding clinicopathological features, and summed up the points to obtain the total points. The predicted 1‐year and 3‐year OS can be estimated based on the total points of each patient.

**Figure 3:**
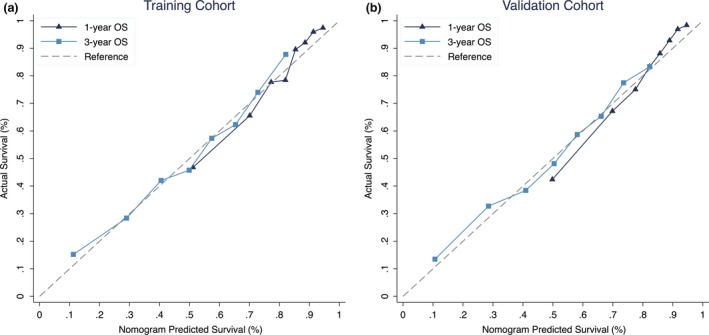
Calibration plots suggested that the predicted 1‐year and 3‐year OS were in agreement with the actual OS in the (A) training and (B) validation cohort.

A nomogram was developed using the training cohort (Fig. 2). Each prognostic factor used to create the nomogram was given a score. By adding up these scores, we can calculate a total score. Then we can draw a straight line down from the total point scale to estimate the 1‐year and 3‐year OS. The C‐index of the nomogram were 0.722 (95% CI 0.710–0.734) and 0.725 (95% CI 0.713–0.736) in the training and validation cohort, respectively. The C‐index of the nomogram is higher than ER status, PR status, HER2 status, and the presence of liver/lung/brain metastases (Table S2). The calibration plots suggested that the accuracy of the predicted 1‐year and 3‐year OS using the nomogram is excellent in both the training and validation cohorts (Fig. 3).

### Risk stratifications using the new nomogram

The distribution of the predicted 1‐year and 3‐year OS was shown in Figure S1. We assigned the patients into different subgroups based on the quartile of the predicted 3‐year OS. Using our model to stratify patients with only bone metastases and ER+/HER2‐ diseases into four different groups allowed for significant distinctions between the KM curves (Figure S2). The first, second, third, and fourth quartile subgroups had 98.2%, 93.5%, 80.1%, and 68.0% 1‐year OS, and 82.2%, 63.3%, 49.6%, and 25.4% 3‐year OS, respectively. Similarly, in patients with visceral metastases (lung, liver, or brain) and ER‐/PR‐ disease (*N* = 5 patients in the first quartile group and were excluded), three subgroups of patients also had significant distinctions among the KM curves (Figure S2). The second, third, and fourth quartile subgroups had 98.1%, 84.0%, and 52.1% 1‐year OS, and 71.1%, 44.6%, and 19.0% 3‐year OS, respectively.

### Exploratory analysis: estimation of the survival benefit of local surgery in stage IV breast cancer patients

With the nomogram, we can estimate the survival benefit of surgery for each patient, by calculating the difference of the predicted OS when the patient was considered as received and did not receive surgery. The median (25–75th percentile) benefit of surgery of 1‐yr and 3‐yr OS was 0.8% (0.3–1.6%) and 1.6% (0.7–3.0%), respectively.

## Discussion

There is an increasing interest in stage IV breast cancer patients, as the proportion of these patients seems to be higher in recent years, when compared with the past. Systemic use of advanced imaging methods, such as PET‐CT scanning, should be the underlying reasons, and this phenomenon was called as stage migration, that is some patients who previously would have been classified as early‐stage, would be reclassified to late‐stage, due to the advanced imaging examinations. The clinical decision‐making for these patients was highly dependent on the predicted survival. However, different metastatic patterns at initial diagnosis (bone, brain, liver lung, etc.) combined with different molecular disease subtypes (HR+/HER2‐, HR+/HER2+, ER‐/HER2+, ER‐/HER2‐) may lead to varied overall survival in these patients 7, 8, 9. Accurately predicting the survival of these patients is of paramount importance. Many RPMs are widely used in early‐stage breast cancer 1; however, there are only a few RPMs that have been reported in advanced/metastatic breast cancer. Lee et al. 12 developed a nomogram to predict the survival time in women with advanced breast cancer using data from clinical trials conducted by the Australia & New Zealand Breast Cancer Trials Group. Prognostic factors used to create that nomogram included ECOG status, ER status, neutrophil levels, age, number of metastatic sites, hemoglobin levels, and alkaline phosphatase levels. Lee et al. 13 used multicenter data from hospitals in South Korea and developed a PMOS system that utilized stage, HR status, Ki67 index, distant metastasis‐free interval, symptoms, and number of metastatic sites to predict the survival of patients with metastatic breast cancer. Both of these studies focused on predicting survival of patients with metastatic breast cancer who present with distant metastatic events a period of time after the treatment for the primary breast tumor. These patients are different from de novo stage IV breast cancer patients, as they are reported to have more favorable outcomes 14. In this study, we used the NCDB database to retrieve data from all stage IV breast cancer patients, developed a nomogram predicting the 1‐year and 3‐year OS rates for these patients.

The value of this nomogram can be seen in the stratification analysis. As per the recommendations from current guidelines 15, hormone receptor‐positive advanced breast cancer patients without visceral crisis should receive endocrine therapy as a first‐line therapy. However, our study showed that from these patients the new nomogram was able to identify a high‐risk subgroup who might need more intensive therapy (e.g., chemotherapy) first. In patients with only bone metastasis and ER+/HER2‐ diseases, the 3‐year OS rates were 82.2% and 25.4% in the first and fourth quartile subgroups, respectively. Therefore, this new model can identify high‐risk patients who were considered to have a favorable prognosis based on the current standards. Similarly, in patients with visceral metastases and ER‐/PR‐ diseases, the new nomogram can also identify low‐risk patients, with 1‐year and 3‐year OS of 98.1% and 71.1%, respectively. Prognosis stratifications using our new model would be informative and helpful for clinical decision‐making. It could inform the risks and benefits of certain treatment plans, aid in designing an appropriate surveillance plan, and provide psychological/sentimental support.

In this study, we noticed that the predicted benefits of local surgery on OS in stage IV breast cancer patients were very low (<2%). This is consistent with the Tata trial 16, in which stage IV breast cancer patients were randomized to locoregional treatment versus no locoregional treatment group with 2‐year OS of 41.9% versus 43.0%, respectively. Similarly, the MF07‐01 trial 17 also revealed that at 54 months, the survival rate was 35% and 31% in the surgery and no surgery group, respectively. In addition, they reported that the benefit of surgery was more significant in patients with bone metastasis only. In contrast, we did not observe any association between the benefit of surgery with any known clinicopathological features. Including treatment variables as predictors may lead to bias, such as confounding by indications. However, we suggested that in real world, whether a treatment was implemented or not may also have prognostic role as well. Some patients, even if suitable for surgery or chemotherapy, may refuse the treatments due to some reasons such as insurance coverage or religion belief. These patients may possibly have inferior survival. Thus, we suggested that treatment variables should be included as predictors.

## Limitations

There were several limitations in this study. The first major limitation stemmed from the lack of information concerning the use of endocrine therapy and anti‐HER2 therapy. Different treatment regimens and patient responses to these therapies 7 are possibly strong predictors for OS. Furthermore, effect modifications may exist between these therapies and the ER/PR/Her2 status or metastatic patterns. Therefore, future studies incorporating these predictors may improve our nomogram. Furthermore, lack of information about the metastatic tumor (ER/PR/HER2 status) was also one of the limitations of the nomogram. Second, cancer registry data may be miscoded, which could bring significant bias to our analysis 18. However, the large sample sizes and well represented patient groups offsets many of the disadvantages of these databases. In our study, we cannot distinguish the de novo stage IV breast cancer patients from those who progressed to stage IV after adjuvant therapies. This is a major limitation. Several studies have showed that these two subsets of patients had different survival, and for the latter ones, more variables (disease free interval, adjuvant therapies, etc.) could be incorporated in our nomogram to enhance the performance. More studies are needed. Third, we randomly separate the population into two cohorts (training and validation cohort). We still need another population from different country to externally validate this nomogram. In addition, we need to be aware that the use of our nomogram in populations from randomized clinical trials will be the gold standard of its validation, and observational data are likely to provide misleading estimates of treatment effects 19.

## Summary

In this study, we developed a novel nomogram predicting the 1‐year and 3‐year OS of stage IV breast cancer patients using national cancer database. The new nomogram can stratify patients into different risk subgroups. A prospective, internationally collaborative study is needed to further validate the new nomogram.

## Conflict of Interest

The authors have no conflict of interest to disclose.

## Supporting information


**Figure S1.** We used the kernel density plot to illustrate the distribution of the predicted (A) 1‐year OS and (B) 3‐year OS of our study population.

**Figure S2.** Patients were categorized into four subgroups, based on quartile of their predicted OS. (A) In patients with ER+/HER2‐ and bone metastasis only, the four subgroups of patients had significantly diverged KM curves. (B) In patients with visceral metastasis, ER‐ and PR‐negative diseases, only five were assigned in the first quartile subgroup (Highest predicted OS) and they were excluded for analysis. The remaining subgroups also had significantly diverged KM curves.

**Data S1.** Determine the training and validation cohort.

**Table S1.** Univariate Cox regression analysis.
**Table S2.** C‐index of the nomogram and clinicopathological features.
