# Supplementary figures and images for: Development and validation of a nomogram predicting the overall survival of stage IV breast cancer patients
Source: Cancer Med. 2017 Oct 4;6(11):2586–94. doi: 10.1002/cam4.1224 (PMC5673913; doi:10.1002/cam4.1224)

# Distribution of predicted OS

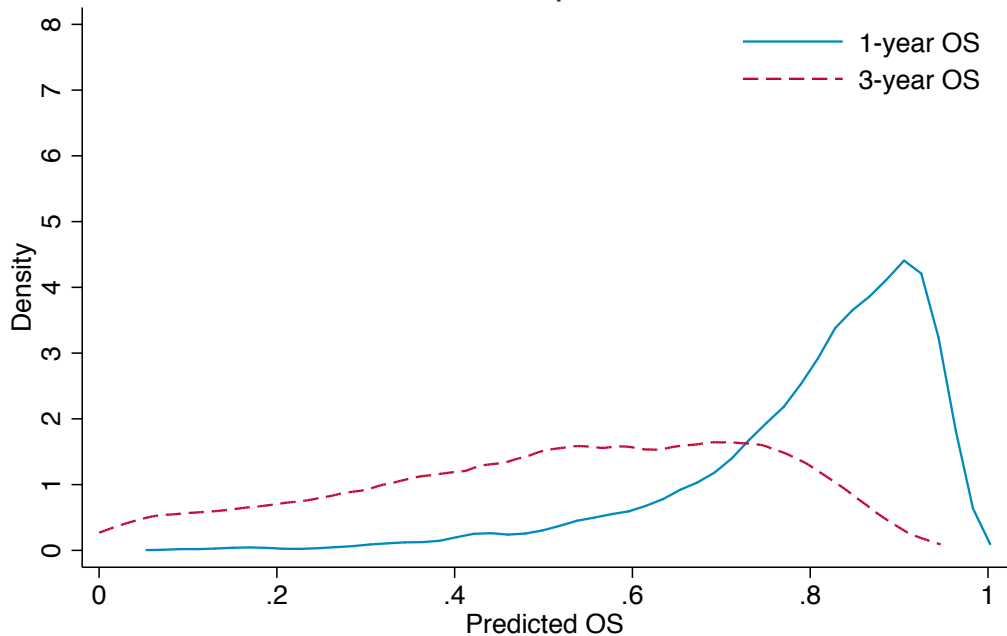

kernel = epanechnikov, bandwidth = 0.0178

Supplement: Supplementary file 1 — Figure S1. We used the kernel density plot to illustrate the distribution of the predicted (A) 1‐year OS and (B) 3‐year OS of our study population. [file CAM4-6-2586-s001.pdf]
