# Supplementary material for: Development and validation of a nomogram predicting the overall survival of stage IV breast cancer patients
Source: Cancer Med. 2017 Oct 4;6(11):2586–94. doi: 10.1002/cam4.1224 (PMC5673913; doi:10.1002/cam4.1224)
Supplement: Supplementary file 2 — Figure S2. Patients were categorized into four subgroups, based on quartile of their predicted OS. (A) In patients with ER+/HER2‐ and bone metastasis only, the four subgroups of patients had significantly diverged KM curves. (B) In patients with visceral metastasis, ER‐ and PR‐negative diseases, only five were assigned in the first quartile subgroup (Highest predicted OS) and they were excluded for analysis. The remaining subgroups also had significantly diverged KM curves. [file CAM4-6-2586-s002.pdf]

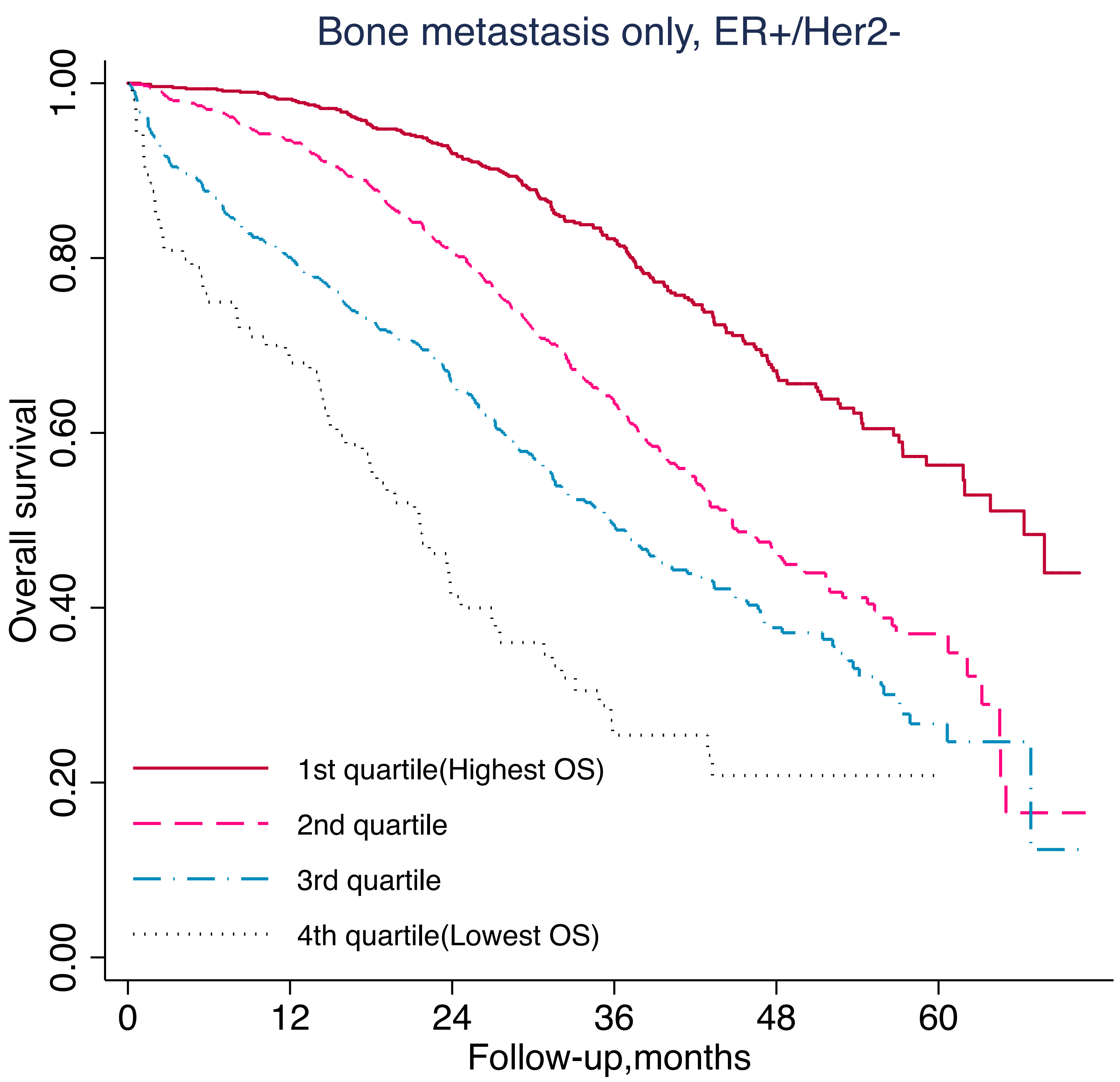

Number at risk

	0	12	24	36	48	60
1st quartile:	776	741	601	393	184	49
2nd quartile:	700	621	463	261	109	19
3rd quartile:	513	384	264	138	69	15
4th quartile:	105	68	33	15	6	0

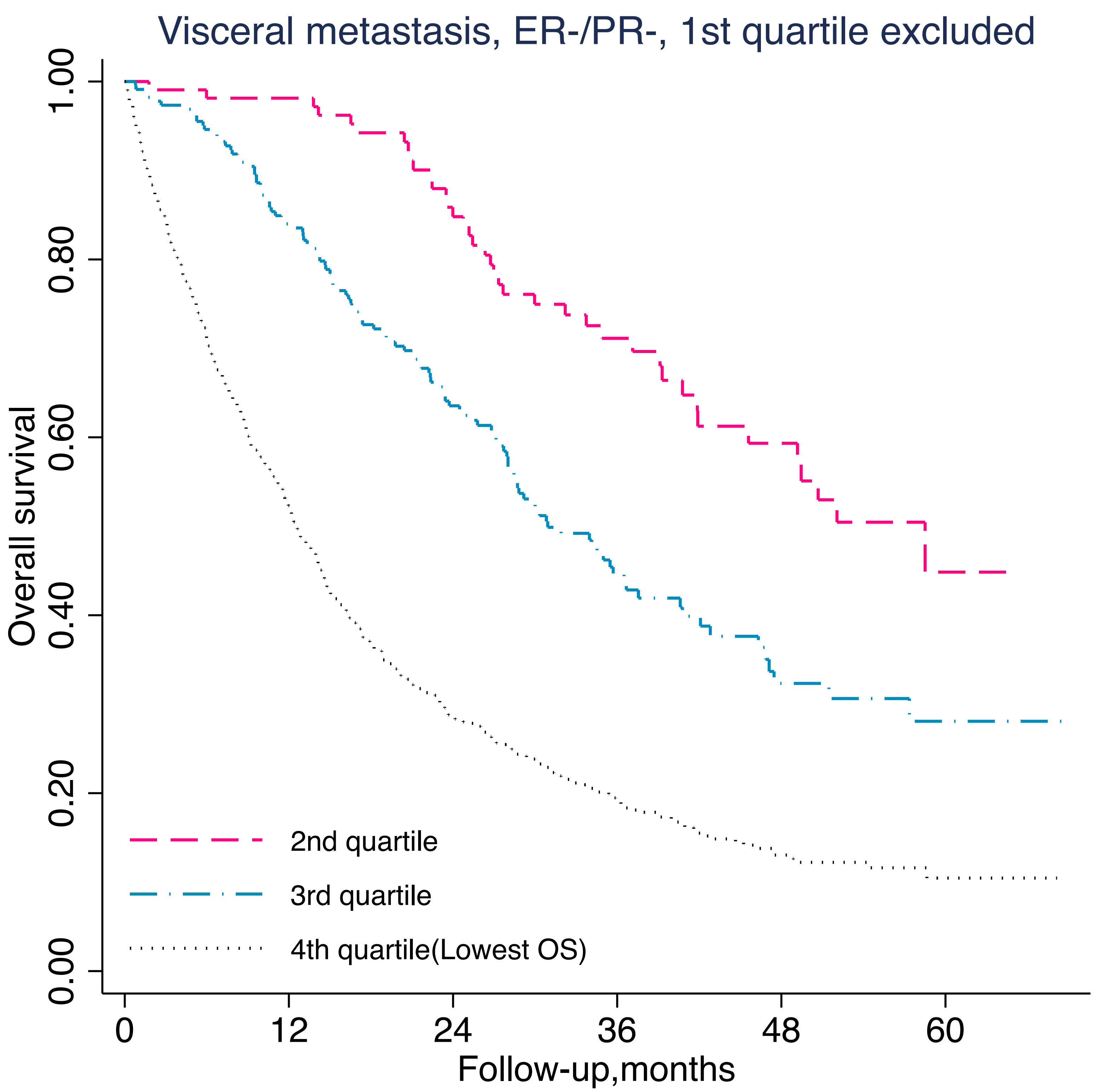

Number at risk

	0	12	24	36	48	60
2nd quartile:	107	103	80	50	28	6
3rd quartile:	226	182	118	52	23	10
4th quartile:	801	398	181	85	34	8
