# Supplementary material for: Development and validation of a nomogram predicting the overall survival of stage IV breast cancer patients
Source: Cancer Med. 2017 Oct 4;6(11):2586–94. doi: 10.1002/cam4.1224 (PMC5673913; doi:10.1002/cam4.1224)
Supplement: Supplementary file 3 — Data S1. Determine the training and validation cohort. [file CAM4-6-2586-s003.docx]

Supplementary File 1

1. Determine the training and validation cohort.

We assigned the patients into the training and validation cohort based on the last number/letter of their PUF_CASE_ID. The PUF_CASE_ID is the unique identifier for each patients in the NCDB. Patients were assigned into the training cohort, if the last number/letter of their PUF_CASE_ID were “0”, “2”, “4”, “6”, “8”, “a”, “c” and “e”. We assigned them into the validation cohort, if the last number/letter of their PUF_CASE_ID were “1”, “3”, “5”, “7”, “9”, “b”, “d” and “f”.
