# Supplementary material for: Development and validation of a nomogram predicting the overall survival of stage IV breast cancer patients
Source: Cancer Med. 2017 Oct 4;6(11):2586–94. doi: 10.1002/cam4.1224 (PMC5673913; doi:10.1002/cam4.1224)
Supplement: Supplementary file 4 — Table S1. Univariate Cox regression analysis. Table S2. C‐index of the nomogram and clinicopathological features. [file CAM4-6-2586-s004.docx]

| Supplementary Table 1, Univariate cox regression analysis | | |
| --- | --- | --- |
| Features | HR(95%CI) | P |
| Race |  |  |
| White | 1 |  |
| African American | 1.41(1.26-1.59) | <0.001 |
| Others | 0.71(0.54-0.95) | 0.02 |
| Age group |  |  |
| <=60 | 1 |  |
| >60 | 1.43(1.30-1.57) | <0.001 |
| Charlson/Deyo Score |  |  |
| 0 | 1 |  |
| 1 | 1.48(1.31-1.67) | <0.001 |
| 2 | 2.25(1.80-2.81) | <0.001 |
| Histology |  |  |
| IDC | 1 |  |
| ILC | 0.89(0.75-1.05) | 0.158 |
| NOS/Others | 1.05(0.92-1.19) | 0.512 |
| T-stage |  |  |
| T0-T1 | 1 |  |
| T2 | 1.23(1.02-1.48) | 0.029 |
| T3 | 1.45(1.18-1.78) | <0.001 |
| T4 | 2.35(1.95-2.83) | <0.001 |
| Tx | 2.36(1.99-2.79) | <0.001 |
| N-stage |  |  |
| N0 | 1 |  |
| N1 | 1.05(0.86-1.28) | 0.638 |
| N2 | 1.18(0.96-1.44) | 0.122 |
| N3 | 1.11(0.91-1.37) | 0.307 |
| Nx | 1.83(1.53-2.18) | <0.001 |
| Grade |  |  |
| I | 1 |  |
| II | 1.11(0.90-1.37) | 0.33 |
| III | 1.60(1.31-1.97) | <0.001 |
| ER |  |  |
| Negative | 1 |  |
| Positive | 0.48(0.43-0.53) | <0.001 |
| PR |  |  |
| Negative | 1 |  |
| Positive | 0.50(0.46-0.55) | <0.001 |
| HER-2 |  |  |
| Negative | 1 |  |
| Positive | 0.68(0.60-0.76) | <0.001 |
| Bone metastasis |  |  |
| No | 1 |  |
| Yes | 0.94(0.85-1.03) | 0.177 |
| Lung metastasis |  |  |
| No | 1 |  |
| Yes | 1.67(1.51-1.85) | <0.001 |
| Liver metastasis |  |  |
| No | 1 |  |
| Yes | 1.55(1.40-1.72) | <0.001 |
| Brain metastasis |  |  |
| No | 1 |  |
| Yes | 2.24(1.89-2.64) | <0.001 |
| Breast Surgery |  |  |
| No_surgery | 1 |  |
| BCS | 0.53(0.46-0.61) | <0.001 |
| Mastectomy | 0.54(0.49-0.60) | <0.001 |
| Radiation therapy |  |  |
| No | 1 |  |
| Yes | 0.71(0.65-0.79) | <0.001 |
| Chemotherapy |  |  |
| None | 1 |  |
| Single-agent Chemotherapy | 1.14(0.98-1.31) | 0.082 |
| Multiagent Chemotherapy | 0.76(0.68-0.84) | <0.001 |
| ER, estrogen receptor; PR, progesterone receptor; HER2, Human epidermal growth factor receptor 2; BCS, Breast-conserving Surgery.IDC, Infiltrating ductal carcinoma, ILC, Infiltrating Lobular carcinoma; NOS, Non otherwise spesific | | |

| Supplementary Table 2, C-index of the nomogram and clinicopathological features | | |
| --- | --- | --- |
| Variable | C-index | 95%CI |
| Training cohort | | |
| Nomogram | 0.722 | 0.710-0.734 |
| ER | 0.589 | 0.577-0.600 |
| PR | 0.595 | 0.583-0.607 |
| HER2 | 0.538 | 0.527-0.548 |
| Liver metastasis | 0.549 | 0.538-0.561 |
| Lung metastasis | 0.556 | 0.545-0.568 |
| Brain metastasis | 0.53 | 0.522-0.537 |
| Validation cohort | | |
| Nomogram | 0.725 | 0.713-0.736 |
| ER | 0.598 | 0.586-0.609 |
| PR | 0.594 | 0.582-0.606 |
| HER2 | 0.529 | 0.518-0.539 |
| Liver metastasis | 0.553 | 0.544-0.566 |
| Lung metastasis | 0.561 | 0.549-0.572 |
| Brain metastasis | 0.532 | 0.526-0.540 |
| ER, estrogen receptor; PR, progesterone receptor; HER-2, Human epidermal growth factor 2; | | |
